# Use of *Trichoderma* culture filtrates as a sustainable approach to mitigate early blight disease of tomato and their influence on plant biomarkers and antioxidants production

**DOI:** 10.3389/fpls.2023.1192818

**Published:** 2023-07-17

**Authors:** Muhammad Imran, Kamal A. M. Abo-Elyousr, Magdi A. A. Mousa, Maged M. Saad

**Affiliations:** ^1^ Department of Agriculture, Faculty of Environmental Science, King Abdulaziz University, Jeddah, Saudi Arabia; ^2^ Department of Plant Pathology, Faculty of Agriculture, University of Assiut, Assiut, Egypt; ^3^ Department of Vegetable Crops, Faculty of Agriculture, Assiut University, Assiut, Egypt; ^4^ DARWIN21, Center for Desert Agriculture, Biological and Environmental Sciences & Engineering Division (BESE), King Abdullah University of Science and Technology (KAUST), Thuwal, Saudi Arabia

**Keywords:** *Alternaria solani*, culture filtrates, antioxidants, volatile metabolites, biocontrol

## Abstract

**Introduction:**

*Alternaria solani* is a challenging pathogen in the tomato crop globally. Chemical control is a rapid approach, but emerging fungicide resistance has become a severe threat. The present study investigates the use of culture filtrates (CFs) of three species of *Trichoderma* spp. to control this disease.

**Methods:**

Highly virulent *A. solani* strain and three *Trichoderma* fungal strains viz., *T. harzianum* (Accession No: MW590687), *T. atroviride* (Accession No: MW590689) and *T. longibrachiatum* (Accession No: MW590688) previously isolated by authors were used in this study. The efficacy of culture filtrates (CFs) to mitigate early blight disease were tested under greenhouse and field conditions, experiments were conducted in different seasons of 2020 using a tomato variety “doucen”.

**Results and discussion:**

The CFs of *T. harzianum*, *T. longibrachiatum*, and *T. atroviride* significantly inhibited the *in vitro* mycelial growth of *A. solani* (62.5%, 48.73%, and 57.82%, respectively, followed by control 100%). In the GC–MS analysis of *Trichoderma* CF volatile compounds viz., harzianic acid (61.86%) in *T. harzianum*, linoleic acid (70.02%) in *T. atroviride*, and hydroxymethylfurfural (68.08%) in the CFs of *T. longibrachiatum*, were abundantly present. Foliar application of CFs in the greenhouse considerably reduced the disease severity (%) in all treatments, viz., *T. harzianum* (18.03%), *T. longibrachiatum* (31.91%), and *T. atroviride* (23.33%), followed by infected control (86.91%), and positively affected the plant biomarkers. In the greenhouse, the plants treated with CFs demonstrated higher flavonoids after 6 days of inoculation, whereas phenolic compounds increased after 2 days. The CF-treated plants demonstrated higher antioxidant enzymes, i.e., phenylalanine ammonia-lyase (PAL) and peroxidase (POD), after 4 days, whereas polyphenol oxidase (PPO) was higher after 6 days of inoculation, followed by healthy and infected controls. In open field conditions, disease severity in CF-treated plants was reduced in both seasons as compared to naturally infected plants, whereas CF-treated plants exhibited a higher fruit yield than controls. The present results conclude that CFs can be a potential biocontrol candidate and a promising alternative to the early blight pathogen for sustainable production.

## Graphical abstract

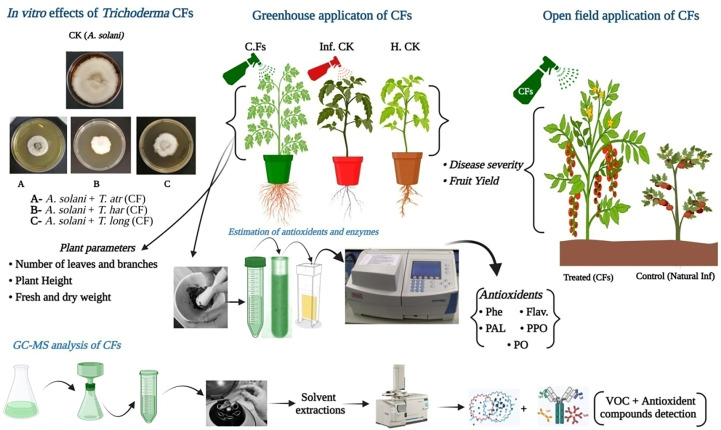

## 1 Introduction

Tomato (*Solanum lycopersicum* L.) is a very significant crop that is widely grown worldwide, including Saudi Arabia (Imran et al., 2021), and is considered a major contributor to the fruit and vegetable diet of humans (Kapsiya et al., 2015). Tomato plants are vulnerable to various biotic factors, including viruses, nematodes, fungus, and bacteria (Imran et al., 2022a), under favorable growth conditions in this region, but the growth yield and production of tomatoes are mainly affected by fungal phytopathogens as various fungal diseases on plants have been reported during different growth stages, which leads to significant pre- and post-harvest yield losses (Gondal et al., 2012; Kolomiets et al., 2017; Tomazoni et al., 2017; González-Fernández et al., 2021; Abo-Elyousr et al., 2022). Among the fungal diseases of tomatoes, early blight disease caused by a pathogen called *Alternaria solani* is one of the most destructive diseases reported worldwide (Koley et al., 2015; Camlica and Tozlu, 2019; Mazrou et al., 2020; Zhang et al., 2020a; Imran et al., 2022b). Due to its adverse effects, this pathogen has drawn great attention over the years due to extensive yield losses in crops (Bauske et al., 2017). Various fungicides of different modes of action have been commonly used to control this disease, but in severe disease outbreaks, multiple applications of fungicides with a higher dose rate are required, which leads to the development of resistance in fungal pathogens (Pasche et al., 2004; Zhang et al., 2017; Zhang et al., 2020b) and ultimately may increase the toxicity of soil, which affects the microbiota population. The development of resistance in fungal pathogens is mainly associated with the detection of point mutations (Senthil et al., 2008; Edin et al., 2019) in the genetic material of fungal pathogens, which aids endurance (Sriwati et al., 2019; Wang W et al., 2021; Zhang et al., 2021). Except for emerging resistance, the multiple applications also pollute the environment, which has a negative impact on human health. Therefore, alternative approaches must be adopted to overcome the resistance problems in the pathogen of early blight disease in tomatoes.

Most recent studies reported the use of various endophytic microorganisms isolated from the rhizospheric zone of plants and screened for their inhibitory antagonistic potential against various fungal pathogens, including *A. solani* (Imran et al., 2022a; Imran et al., 2022b). Among the fungal bioagents, *Trichoderma harzianum*, *T. atroviride*, *T. longibrachiatum*, *T. gamsii*, and *T. asperellum* has been widely reported and used as the best biocontrol candidates for potential antagonistic activity against a variety of phytopathogens (Mazrou et al., 2020; Stracquadanio et al., 2020; Castro-Restrepo et al., 2022; Imran et al., 2022b). *Trichoderma* species as cell suspensions or culture filtrates (by acting as protective barriers) can effectively assist the plant to survive microbial competition as well as environmental stresses (Rahman et al., 2018). The results of a study by Alka et al. represent a significant *in vitro* and *in vivo* reduction of a fungal pathogen, *Rhizopus oryzae*, causing Rhizopus rot of tomatoes (Alka and Prajapati, 2017), when culture filtrates of *Trichoderma* species were applied. Additionally, the culture filtrates of various *Trichoderma* species as biofungicides against various fungal pathogens, viz., *Colletotrichum gloeosporioides* (Nurbailis et al., 2019), against anthracnose of great millet (Manzar et al., 2021), *Pythium* species, and *Phytophthora* species, demonstrated inordinate antifungal potential (Ben M'henni et al., 2022). The use of various *Trichoderma* species has widely been reported as an eco-friendly and safe approach to the control of plant diseases (Alka and Prajapati, 2017; Nurbailis et al., 2019; Manzar et al., 2022). The application of culture filtrates (CFs) of *Trichoderma* species as biocontrol not only acts as biostimulants against the inhibition of pathogens but also induces systemic or localized resistance in plants to biotic stresses and, ultimately, as a growth regulator, increases plant biomass (Abbas et al., 2019; Guzmán-Guzmán et al., 2019). The inhibition of fungal pathogens by *Trichoderma* species implicates various mechanisms, including specific metabolite and phenolic compound production, fibrolytic enzymes, various antimicrobial substances, direct parasitization, and competition for food by nutrients (Cavallo et al., 2020; Dini and Laneri, 2021; Dini et al., 2021a; Iannaccone et al., 2022). Moreover, *Trichoderma* species stimulate the production of phenolic compounds, which increase the nutraceutical value and defense system of plants (Cavallo et al., 2020; Dini and Laneri, 2021), causing the degradation of polysaccharides, chlorophenols, hydrocarbons, and xenobiotic pesticides (Zafra and Cortés-Espinosa, 2015; Dini et al., 2021b). During infection by a pathogen, *Trichoderma* species activate systemic resistance through multiple hormonal signaling pathways, which act as the primary barrier to the plant defense system (Mendoza-Mendoza et al., 2018). In the defense system of plants, the activation of defense enzymes, viz., polyphenol oxidase (PPO), peroxidase (POD), phenylalanine ammonia lyase (PAL), chitinase, β-1,3-glucanase, and various antioxidants such as catalase, flavonoids, and phenolics, has a significant role in inducing resistance (Anand et al., 2007; Almagro et al., 2009), along with microbial volatile elicitor compounds (Kashyap et al., 2022). Various studies reported a significant increase in the production of defense enzymes following the application of culture filtrates and/or cell suspensions of *Trichoderma* species to plants (Akram et al., 2021; Heflish et al., 2021; Mahmoud et al., 2021; Tripathi et al., 2021; Vukelić et al., 2021). Phenolic compounds are well known as they contain a wide group of chemicals (phenocarbonic acids, flavonoids, phenolic acids, lignans, polymeric lignans, and stilbenes) and, as plant growth regulators, modulate physiological processes such as vesicle trafficking, membrane permeability, signal transduction, and gene transcription in plants (Quideau et al., 2011; Babenko et al., 2019). Generally, phenylalanine ammonia lyase (PAL) has an imperative role in the biosynthesis of phenolic compounds as it catalyzes the non-oxidative eradication of the -NH_2_ group from L-phenylalanine (Phe) to construct trans-cinnamate, which is a starting molecule for the synthesis of other phenolic compounds (Pereira et al., 2009; Jun et al., 2018). While polyphenol oxidase (PPO) catalyzes the O_2_-dependent oxidation of ortho (o)-phenolics to o-quinones, which condensed the nutritive importance of protein (Constabel and Barbehenn, 2008). Peroxidase (POD) utilizes O_2_ or H_2_O_2_ to oxidize various molecules, which are used in diagnosis and immune assays (Yoshida et al., 2003). The induction of defense enzymes, antioxidants, phenols, and flavonoids is mainly responsible for the activation of the defense system in plants, which protects them from the infection of pathogens (Schulz-Bohm et al., 2017; Zehra et al., 2017; Kaur et al., 2022). Previously, the effect of foliar application of *Trichoderma* as cell suspension on disease severity and fruit yield under greenhouse and open field conditions was studied (Imran et al., 2022a; Imran et al., 2022b). Considering the emerging fungicide resistance risks and their impact on the environment, some eco-friendly approaches are needed to adopt for the control of early blight pathogen, and to the author’s knowledge, no study so far has been conducted to use the culture filtrates (CFs) of *Trichoderma* for the control of early blight disease in this region. Therefore, the aim of the present study was to investigate the effect of foliar application of *Trichoderma* culture filtrates (CFs) in greenhouses on disease severity and plant biomass; to study the effect of CFs on defense enzymes and antioxidant production in tomato plants; and to study the effect of CFs in open fields under natural infection conditions and their influence on fruit yield.

## 2 Materials and methods

### 2.1 Collection of the fungal pathogen, bioagents, and growth conditions

A highly virulent *A. solani* strain was previously isolated, screened, and identified by Imran et al. (2022b) was collected from the fungal stock culture of the Laboratory of Plant Pathology, Department of Agriculture, King Abdulaziz University, Jeddah, Saudi Arabia. The pathogenic strain was subcultured on a potato dextrose agar (PDA) medium plate at 27°C for 5–7 consecutive days, and the actively grown strain was preserved at 4°C for further use.

Three *Trichoderma* fungal strains, viz., *T. harzianum* (Accession No.: MW590687), *T. atroviride* (Accession No.: MW590689), and *T. longibrachiatum* (Accession No.: MW590688) previously isolated and identified by Imran et al. (2022a), were obtained from the fungal stock culture of the Laboratory of Plant Pathology, Department of Agriculture, King Abdulaziz University, Jeddah, Saudi Arabia. The *Trichoderma* strains were subsequently subcultured on PDA medium plates at 27°C for 3 days, and the active colony culture was preserved at 4°C.

### 2.2 *In vitro* assay

#### 2.2.1 Preparation of culture filtrates and mycelial growth inhibition

Culture filtrates of *Trichoderma* strains were initially prepared by transferring 5-mm mycelial discs (3-day-old previously grown on PDA) to 50 ml sterile conical flasks containing 25 ml potato dextrose broth (PDA lacking agar). Mycelial discs of uniform diameter (8–10 discs/flask) were carefully placed (in floating positions) in PDB containing flasks that were subsequently incubated at 27°C in a shaker (125×*g*) for 7–10 days. The resulting suspension was centrifuged (10,000×*g* for 10 min at 4°C) and filtered through Whatman No. 1 filter paper (Sigma-Aldrich, USA), followed by a final filtration through a Manifolds vacuum filtration unit (MFA-1S SS316L, 100 µm; Bioevopeak, Shandong, China) for the purity of culture filtrates. The obtained supernatant (100% purity) was preserved at 4°C.

To assess *in vitro* mycelial growth inhibition, previously prepared culture filtrates (CFs) of *Trichoderma* strains were supplemented with PDA medium to evaluate their effect on *A. solani*. Briefly, PDA plates supplemented with culture filtrate (CF) suspension containing 20 ml of PDA/petri plate (1 ml of CF suspension was thoroughly mixed with 9 ml of PDA) were inoculated with a 5-mm mycelial disc (placed face-down in the middle) excised from the edge of a 5-day-old *A. solani* culture previously grown on PDA. Four replicates were used for each strain, whereas six plates were used as a replicate. The plates lacking culture filtrate suspension were used as a control. Plates were incubated at 27°C for 7–10 days until the completion of the control plate with the growth of the pathogen. Then, the colony diameter was measured on each plate and compared with the control.

### 2.3 Assessment and characterization of volatile compounds in fungal bioagents by gas chromatography–mass spectrometry analysis

#### 2.3.1 Extraction of metabolites from culture filtrates

Metabolites from the culture filtrates were extracted with the method described by Stracquadanio et al. (2020). Briefly, the culture filtrates of *Trichoderma* strains were extracted three times by using ethyl acetate solvent (1:1). To dry the combined organic fraction, magnesium sulfate (MgSO_4_) was used and evaporated at 35°C under reduced pressure. The residues having a red-brown color were recovered and dissolved in 10% methanol (CH_3_OH) or dimethylsulfoxide (DMSO), and extracts were stored at −20°C to perform further analysis.

#### 2.3.2 GC–MS analysis

The metabolites obtained from the culture filtrates of selected fungal bioagents were used to perform GC–MS analysis for the detection of active biomolecule components. The volatile compounds in fungal metabolites were identified with a single quadrupole mass spectrometer (GC–MS) detector (Stoppacher et al., 2010; Siddiquee et al., 2012). The identification of the volatile compounds in the culture filtrate of *Trichoderma* species was conducted by GC–MS analysis. This chemical identification of the compounds was determined by injecting the standard compounds into GC–MS or by comparison to library mass spectra from the NIST database. The energy for electron impact was 70eV, whereas the ion source temperature was adjusted to 250°C. The electron impact (EI) mass scan (m/s) range was 40–450 Da in fully scan acquisition mode (Hernádez-Rodrίguez et al., 2008; Khan et al., 2021). The spectra of the identified compounds were compared with the available spectra of compounds in the GC–MS of the National Institute of Standards and Technology (NIST) database. More than 90% resemblance was considered a threshold for detection.

### 2.4 In planta assay

#### 2.4.1 Effect of culture filtrates on disease severity

To study the efficacy of culture filtrates (CFs) to mitigate early blight disease, experiments were conducted in different seasons of 2020 under the greenhouse using the tomato variety “doucen.” Briefly, tomato seedlings were grown in 18-cm plastic pots containing peat moss (1:3), and at three- to four-leaf stage, seedlings were transplanted to the new pots containing an identical amount of growth medium. Previously prepared culture filtrates (100%) of *Trichoderma* strains were used as foliar application after twelve days of transplanting (30 ml plant^−1^), whereas, after 24 h, the cell suspension of a virulent pathogenic strain (previously grown on PDA for 7 days adjusted with a hemocytometer) was sprayed (10^4^ spores/ml; 30 ml plant^−1^). Plants sprayed with sterile distilled water were treated as healthy controls whereas plants inoculated with the suspension of pathogens were treated as infected controls. Plants were covered with sterile polythene bags for three days to retain humidity for the initiation of infection by pathogens. The humidity (75%–80%) and optimal temperature (27 ± 1°C) inside the greenhouse were maintained. The experiment was performed with six replicates for each treatment, and six plants were subjected to each replicate. The experiment was repeated twice, and disease severity was measured with a reported 0–5 disease rating scale as follows: 0—[no infection on leaves]; 1—[0%–5% infection on leaves]; 2—[6%–20% infection on leaves]; 3—[21%–40% infection on leaves]; 4—[41%–70% infection on leaves], and 5—[>70% infection on leaves] (Gondal et al., 2012). Disease severity (%) was recorded as: disease severity = Σ (no. of infected plants × no. scale)/total no. of plants × higher no. scale × 100 (Akhtar et al., 2016).

#### 2.4.2 Effect of culture filtrates on plant biomass

The biomass of tomato plants, viz., plant height and fresh and dry weight of roots and shoots, were measured after the determination of disease severity. Plant height was calculated inside the greenhouse within the pots, and subsequently, plants were harvested to measure the fresh weight. Then, the plants were placed in a moisture dryer chamber at 60°C for 5 days to ensure the complete drying of the moisture contents. The dry weight of the plants was calculated, and the means were compared among the treatments.

### 2.5 Effect of culture filtrates on secondary metabolite production

#### 2.5.1 Preparation of leaf aliquot and estimation of secondary metabolites

In the greenhouse experiment, leaf samples from each treatment were randomly collected from randomly selected replicates at 0, 2, 4, 6, and 8 days after inoculation. Samples were immersed in liquid nitrogen to obtain the fine powder that was stored at −80°C to measure the secondary metabolites. viz., total phenol and flavonoids contents.

##### 2.5.1.1 Total phenol contents

Total phenol contents in the samples were assayed by using the Folin–Ciocalteu reagent with the method of Meda et al. (2005) with trivial modifications. Briefly, 1 g of powdered sample was dissolved in 5 ml of methanol (80%). The aliquot was centrifuged at 10,000 rpm for 10 min at 4°C. The supernatant was transferred to new 2 ml tubes, followed by storage at −80°C before further analysis. Subsequently, 100 µl of the methanol-extracted sample was assorted with 750 µl of 1 N Folin–Ciocolteu reagent (1:10), followed by incubation for 5 min at room temperature. Thereafter, 60 µl of sodium carbonate (Na_2_CO_3_) (7.5%) was added, and the mixture was incubated for 30 min at room temperature. The absorbance of the mixture was recorded at 750 nm using a UV–Vis spectrophotometer. The phenolic contents in the reaction mixture were determined by using a standard curve obtained from gallic acid (0–5 mg) and expressed as mg/g plant material. Six replicates were used for each treatment.

##### 2.5.1.2 Flavonoid contents

Flavonoid contents in the sample were determined according to the method of Chang et al. (2002) using an aluminum chloride (AlCl_3_) reagent. Briefly, 250 μl of the methanol-extracted samples were mixed into 75 μl sodium nitrite (5%) followed by the addition of 1,250 μl distilled H_2_O followed by incubation for 5 min at room temperature. Afterward, 150 μl aluminum chloride (10%), 500 μl sodium hydroxide (1M), and 275 μl distilled H_2_O were added to the reaction mixture. The mixture was incubated for 5 min at room temperature, and the absorbance was recorded at 510 nm and expressed as mg/g plant material. Six replicates were used for each treatment.

### 2.6 Antioxidant enzyme assay

#### 2.6.1 Protein assays

The concentration of protein in leaf extract was determined by Bradford’s (1976) method using bovine serum albumin (BSA) as a standard. Protein contents were determined by a UV–Vis spectrophotometer (Thermo Fisher Scientific™) at an absorbance of 595 nm, and a standard curve was developed. The standard curve was used to determine secondary metabolites and antioxidants in leaf extract.

##### 2.6.1.1 Preparation of crude enzyme

To prepare crude enzymes, previously ground (powder) leaf tissues were used to estimate antioxidant enzymes, viz., phenylalanine ammonia-lyase (PAL), peroxidase (POD), and polyphenol oxidase (PPO). Briefly, 500 mg of fine powder from each replicate was suspended in 2 ml of extraction buffer containing 20 ml of phenylmethyl sulfonyl fluoride (1 mM) and polyvinylpyrrolidone (0.1%). To determine PAL activity, sodium borate buffer (0.1 M, pH 8.7) was used, while for PO and PPO activity, sodium phosphate buffer (0.1 M, pH 7.0) was used. The extraction was conducted at 4°C, and samples were centrifuged at 12,000×*g* for 20 min at 4°C. The supernatant (the crude enzyme source) was transferred to a new centrifuge tube and preserved at −80°C to determine the enzymatic assay.

##### 2.6.1.2 Phenylalanine ammonia-lyase assay

Phenylalanine ammonia-lyase **(**PAL) activity in treated tomato leaves was determined according to the method of Dickerson et al. (1984). Briefly, the reaction containing 200 µl of crude enzyme extract, 1,300 µl sodium borate buffer (0.1 M, pH 8.7) and 500 µl of L-phenyl alanine (12 mM) was incubated at 37°C in a water bath for 30 min. Subsequently, the PAL activity was determined at 290 nm absorbance with a UV–Vis spectrophotometer, whereas cinnamic acid (0–5 mg, nM) was used as a standard, and the PAL activity was expressed as min/g protein. Six replicates were used for each treatment.

##### 2.6.1.3 Peroxidase assay

Peroxidase (POD) activity in treated tomato leaves was assayed using the method of Hemeda and Klein (1990). Briefly, the substrate was prepared by dissolving 5 ml of H_2_O_2_ (0.3%) in 5 ml of guaiacol (1%) and then adding 50 ml of sodium phosphate buffer (0.05 M, pH 6.5). The reaction mixture was prepared by suspending 1,475 µl of the substrate in 25 µl of crude enzyme extract. The change in absorption was recorded at the absorbance of 470 nm, and PO activity was calculated by the increase in absorbance caused by the oxidation of guaiacol. PO activity was expressed as µmol/min/mg of protein (E = 26.6/mM/cm).

##### 2.6.1.4 Polyphenol oxidase assay

Polyphenol oxidase (PPO) activity was determined using the method of Kumar and Khan (1982). The reaction mixture containing 250 µl crude enzyme extract, 1,000 µl sodium phosphate buffer (0.1 M, pH 6.5), and 500 µl catechol (0.1 M) was incubated at room temperature for 10 min. Then, 500 µl of H_2_SO_4_ (2.5N) was added to stop the reaction. The purpurogallin-formed absorption was observed at an absorbance of 495 nm. While the initial addition of H_2_SO_4_ to the reaction mixture was used as a blank, PPO activity was calculated and expressed as U/min/mg protein (U = change in 0.1 absorbance/min/mg protein).

### 2.7 Open field trails

The efficiency of *Trichoderma* culture filtrates in two consecutive seasons [early (January–March) and late (September–December)] of 2020 was determined in open fields under natural infection conditions. The experimental site was prepared at the “Hada Al Sham” field station of King Abdulaziz University. In the early season, seedlings of the tomato variety “Doucen” were grown in a plastic seedling growing tray (50 holes) containing peat moss (1:3). At the three- to four-leaf stage, seedlings were transplanted in an agricultural field previously prepared (maintaining a 60-cm distance between rows and 45 cm within plants) (**Supplementary Figure 1**). Culture filtrates of fungal bioagents (previously prepared) were used as foliar applications. After one week of transplanting, the suspension of culture filtrates (100%) was applied as a foliar spray (50 ml/plant) to tomato plants. For healthy control, the plants were sprayed with sterile distilled water, whereas the untreated plants (lacking any of the treatment) were subjected to infected control. All treatments were applied in the evening, and plants were left for natural infection in an open field. Plants were irrigated properly as per requirements with a drip irrigation system, and standard agronomic practices were carried out throughout the experiment. For each treatment, including control, plants were randomly selected, and disease severity was recorded with a reported disease rating scale as previously mentioned (Gondal et al., 2012; Ismail et al., 2016). Fruit yield was recorded by harvesting the ripened fruit regularly from all replicates of all treatments, and the total yield for each treatment was calculated and compared.

The experiment was conducted in a completely randomized block design with six replicates, each containing nine plants. All recommended agronomic practices were adopted in the experimental field, and each treatment was applied randomly to plants. An experiment with identical parameters was performed in the late season. Disease severity and fruit yield in both seasons were recorded and compared with controls to observe the efficacy of culture filtrates against the natural infection of the early blight pathogen.

### 2.8 Statistical analysis

All *in vitro* experiments were conducted in triplicate, while field experiments were conducted in quadruplicate. Experiments were performed in a complete randomized design, and all collected data was analyzed by using Statistix 8.1 (Analytical Software, Statistix; Tallahassee, FL, USA, 1985–2003). The data from disease severity was transformed into arcsine values, and a one-way analysis of variance (ANOVA) was performed. The means of replicates in all treatments were compared using Fisher’s least significant difference test at *p* = 0.05 (Steel et al., 1996).

## 3 Results

### 3.1 *In vitro* mycelial growth inhibition by culture filtrates

**Figure 1:**
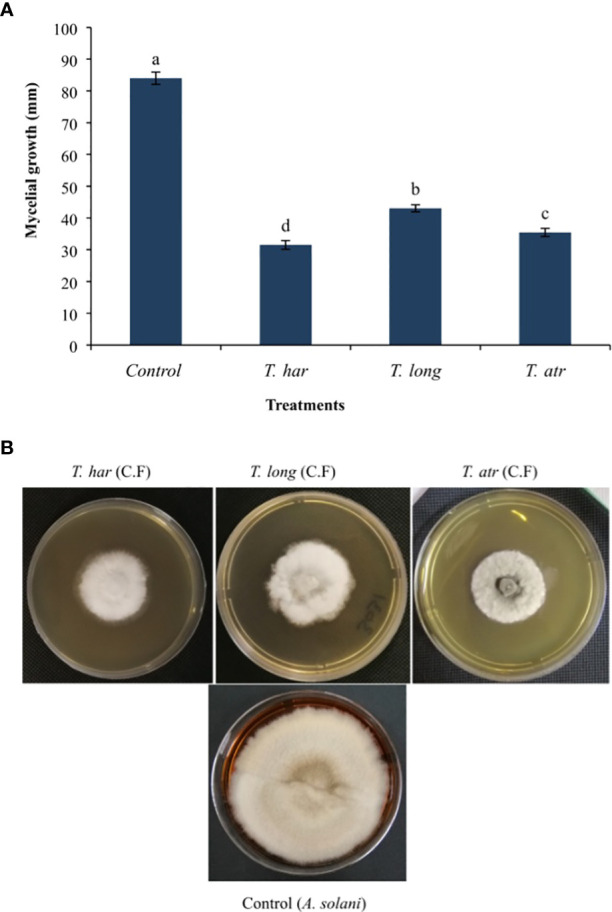
**(A)** *In vitro* effect of *Trichoderma* culture filtrates (CFs) on the mycelial growth of the *A. solani* pathogen. Values followed by different letters indicate that means are significantly different from each other according to Fisher’s least significant difference at *p* = 0.05. *T. har*, *Trichoderma harzianum*; *T. long*, *Trichoderma longibrachiatum*; *T. atr*, *Trichoderma atroviride*. **(B)** The *in vitro* effect of *Trichoderma* culture filtrates on mycelial growth inhibition of the *A*. *solani* pathogen on PDA medium.

The *in vitro* application of *Trichoderma* culture filtrates (CFs) demonstrated significant (*p* = 0.05) mycelial growth suppression of the *A. solani* pathogen. However, the CFs of *T. harzianum* suppressed (62.5%) mycelial growth as lower (31.5 mm) mycelial growth was recorded, followed by the control (84 mm). Meanwhile, mycelial growth inhibited by CFs of *T. longibrachiatum* and *T. atroviride* (48.7% and 57.8%, respectively) was relatively lower than that of *T. harzianum* followed by the control (**Figure 1A**). Apparently, the *in vitro* application of all *Trichoderma* CFs significantly altered the growth pattern of the *A. solani* colony, followed by the control (**Figure 1B**). The results herein presented indicate that even lower volumes (1 ml of CFs to 9 ml of PDA) of CFs of these *Trichoderma* strains showed better growth inhibition of the *A. solani* pathogen.

### 3.2 GC–MS analysis of *Trichoderma* culture filtrates

**Figure 2:**
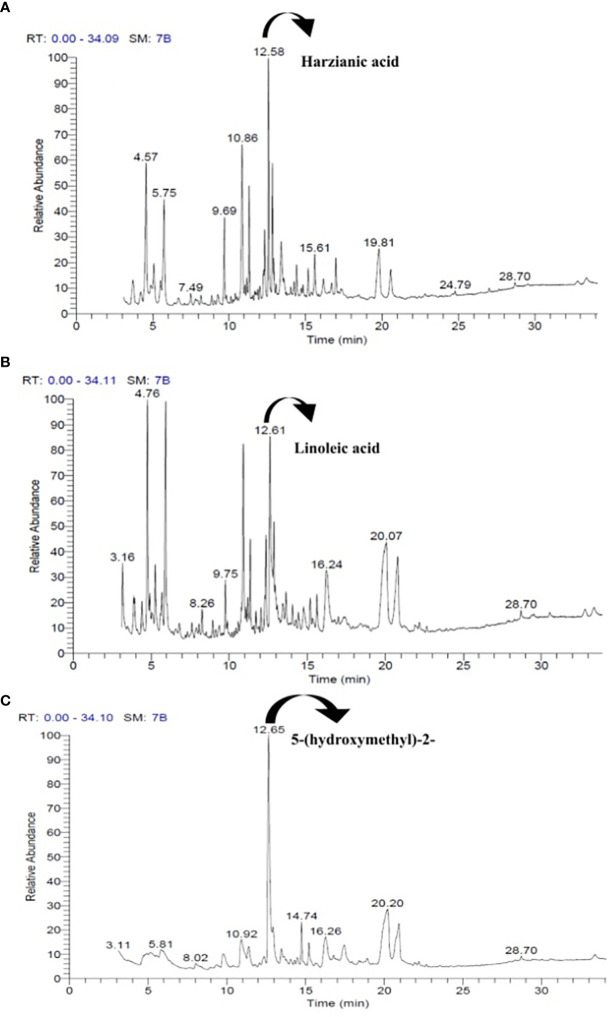
The standard GC–MS chromatograms of *T. harzianum* **(A)**, *T. atroviride* **(B)**, and *T. longibrachiatum* **(C)** strains and the peaks of volatile organic compounds (VOCs) detected from their culture filtrates.

The GC–MS chromatogram of *T. harzianum* demonstrated the presence of 10 peaks (**Figure 2A**) with 27 volatile compounds (**Table 1A**), whereas 25 volatile compounds (**Table 1B**) with eight peaks (**Figure 2B**) in *T. atroviride* and nine peaks (**Figure 2C**) with 32 volatile compounds (**Table 1C**) were detected in *T. longibrachiatum*.

In the CFs of *T. harzianum*, various groups of chemical compounds, viz., aldehydes, hydroxyls, different acids, glycerin, esters, and many hydroxyl groups containing compounds, were detected. The most abundant compound (61.86%) present in CFs of *T. harzianum* was harzianic acid (C_19_H_27_NO_6_), detected at a retention time (Rt) of 12.58 (**Table 1A**; **Figure 2A**). Glycerin was also observed in a higher amount (9.98%), followed by other compounds that ranged between 5% and 8%. The other prominent constituents in the CFs of *T. harzianum* were dl-glyceraldehyde (8.21%), 2-Propanone, 1,3-dihydroxy (6.23%), 3-Deoxy-d-mannoic lactone (5.34%), 1,2,3-Propanetriol, monoacetate (5.10%), 4H-Pyran-4-one,2,3-dihydro-3,5-dihydroxy-6-methyl- (4.94%), and D-Alanine, N-propargyloxycarbonyl-,isohexyl ester (4.41%), whereas other detected compounds were in lower amounts (%).

**Table 1A:** Volatile organic compounds (VOCs) obtained from the culture filtrates of *T. harzianum* strain identified by GC–MS analysis. The bold letters indicates the compound which is abundantly present in the culture filtrates of *T. harzianum*.

Retention time (RT)	Compound name	Structure	MW	Abundance (%)
4.56	dl-Glyceraldehyde	C_3_H_6_O_3_	90	8.21
4.87	Pilocarpine	C_11_H_16_N_2_O_2_	208	0.43
5.08	2-Furanmethanol	C_5_H_6_O_2_	98	1.99
5.51	o-Acetyl-L-serine	C_5_H_9_NO_4_	147	0.98
5.75	2-Propanone, 1,3-dihydroxy	C_3_H_6_O_3_	90	6.23
6.69	Octadecanedioic acid	C_18_H_34_O_4_	314	0.55
7.49	2-Furancarboxaldehyde, 5-methyl-	C_6_H_6_O_2_	110	0.76
9.69	D-Alanine, N-propargyloxycarbonyl-,isohexyl ester	C_13_H_21_NO_4_	255	4.41
10.12	9,12,15-Octadecatrienoic acid,2,3-dihydroxypropyl ester, (Z,Z,Z)-	C_21_H_36_O_4_	352	0.34
10.41	5-Octadecenal	C_18_H_34_O	266	0.50
10.86	Glycerin	C_3_H_8_O_3_	92	9.98
11.16	Tetraacetyl-d-xylonic nitrile	C_14_H_17_NO_9_	343	0.70
11.32	4H-Pyran-4-one,2,3-dihydro-3,5-dihydroxy-6-methyl-	C_6_H_8_O_4_	144	4.94
11.68	2-Cyclohexylpiperidine	C_11_H_21_N	167	0.41
12.33	2-Pentenoic acid, 3-methyl-, methylester	C_7_H_12_O_2_	128	3.87
**12.58**	**Harzianic acid**	**C_19_H_27_NO_6_**	**365.40**	**61.86**
12.84	1,2,3-Propanetriol, monoacetate	C_5_H_10_O_4_	134	5.10
12.92	Maltol	C_6_H_6_O_3_	126	1.14
13.41	Stevioside	C_38_H_60_O_18_	804	3.20
14.03	2-Myristynoyl pantetheine	C_25_H_44_N_2_O_5_S	484	0.39
15.61	4-Amino-1,5-pentandioic acid	C_7_H_13_NO_4_	175	2.28
16.99	7-Oxo-2-oxa-7-thiatricyclo[4.4.0.0(3,8)]decan-4-ol	C_8_H_12_O_3_S	188	1.98
19.81	3-Deoxy-d-mannoic lactone	C_6_H_10_O_5_	162	5.34
20.58	9-Octadecenoic acid (2-phenyl-1,3-dioxolan-4-yl)methyl ester, cis-	C_28_H_44_O_4_	444	1.89
24.79	Ethyl iso-allocholate	C_26_H_44_O_5_	436	0.23
28.70	Hexadecanoic acid,1-(hydroxymethyl)-1,2-ethanediyl ester	C_35_H_68_O_5_	568	0.33
30.05	Ethyl iso-allocholate	C_26_H_44_O_5_	436	0.03

The compound in the bold letter was detected at the highest percentage. Mol, molecular; (*n* = 27).

Various groups of chemical compounds, including methyl, esters, sugar, glycerin, compounds containing an acetyl group, and compounds with a hydroxyl group, were detected in the CFs analysis of *T. atroviride*. The most significant and abundant compound (70.02%) in *T. atroviride* was 9,12-octadecadienoic acid (Z,Z)—which is commonly known as “linoleic acid” at the retention time (Rt) of 12.61 (**Table 1B**; **Figure 2B**). The chemical compounds 3-deoxy-d-mannoic lactone (10.15%), 2-propanone, 1,3-dihydroxy- (8.66%), dl-glyceraldehyde (8.67%), and glycerin (7.2%) were present, whereas the other chemical compounds were present in a lower amount <5%.

The results of GC–MS analysis of *T. longibrachiatum* culture filtrates (CFs) demonstrated the presence of various groups, viz., nitrile, methyl, hydroxyl groups, monosaccharides, glycosides, alcohols, ketoses, and some unknown compounds. The compound 2-furancarboxaldehyde,5-(hydroxymethyl)—commonly known as HMF or 5-(hydroxymethyl)-2—was detected at a higher level (68.08%) at the retention time (Rt) of 12.65 (**Table 1C**; **Figure 2C**). The compounds 3-deoxy-d-mannoic lactone (18.94%) and à-D-glucopyranoside,O-à-D-glucopyranosyl-(1.fwdarw.3)-á-D-fructofuranosyl (5.78%) were present in higher amounts, whereas other compounds were also detected in subordinate amounts (<5%). In these results, the CFs of these *Trichoderma* demonstrated the presence of various chemical compounds in higher amounts, including some common hydroxyl group-containing compounds (**Tables 1A**–**C**).

**Table 1B:** Volatile organic compounds (VOCs) obtained from the culture filtrates of *T. atroviride* strain identified by GC–MS analysis. The bold letters indicates the compound which is abundantly present in the culture filtrates of *T. atroviride*.

Retention time (RT)	Compound name	Structure	MW	Abundance (%)
3.16	2-Propanone, 1-hydroxy-	C_3_H_6_O_2_	74	2.37
3.51	Tetraacetyl-d-xylonic nitrile	C_14_H_17_NO_9_	343	0.25
3.88	1-Butanol, 2-nitro-	C_4_H_9_NO_3_	119	1.06
3.94	Butanedioic acid, 2,3-bis(acetyloxy)-,[R-(R*,R*)]	C_8_H_10_O_8_	234	0.92
4.76	dl-Glyceraldehyde	C_3_H_6_O_3_	90	8.67
5.06	Cyclopropanetetradecanoic acid,2-octyl-, methyl ester	C_26_H_50_O_2_	394	0.48
5.26	2-Furanmethanol	C_5_H_6_O_2_	98	2.35
5.59	Deoxyspergualin	C_17_H_37_N_7_O_3_	387	0.43
5.69	o-Acetyl-L-serine	C_5_H_9_NO_4_	147	1.80
5.93	2-Propanone, 1,3-dihydroxy-	C_3_H_6_O_3_	90	8.66
7.90	Tetraacetyl-d-xylonic nitrile	C_14_H_17_NO_9_	343	0.51
8.26	Cyclopropanetetradecanoic acid,2-octyl-, methyl ester	C_26_H_50_O_2_	394	0.93
8.80	2-Myristynoyl pantetheine	C_25_H_44_N_2_O_5_S	484	0.07
9.75	D-Alanine, N-propargyloxycarbonyl-,isohexyl ester	C_13_H_21_NO_4_	255	2.09
10.44	Dodecanoic acid, 3-hydroxy-	C_12_H_24_O_3_	216	0.26
10.91	Glycerin	C_3_H_8_O_3_	92	7.22
11.36	4H-Pyran-4-one,2,3-dihydro-3,5-dihydroxy-6-methyl-	C_6_H_8_O_4_	144	2.83
11.72	2-Cyclohexylpiperidine	C_11_H_21_N	167	0.92
12.36	2-Pentenoic acid, 3-methyl-, methyl ester	C_7_H_12_O_2_	128	3.52
**12.61**	**9,12-Octadecadienoic acid (Z,Z)-**	**C_18_H_32_O_2_**	**280**	**70.02**
13.09	O-à-D-glucopyranosyl-(1➔3)-á-D-fructofuranosyl	C_18_H_32_O_16_	504	0.24
16.21	d-Mannose	C_6_H_12_O_6_	180	3.82
20.07	3-Deoxy-d-mannoic lactone	C_6_H_10_O_5_	162	10.15
28.70	Ethyl iso-allocholate	C_26_H_44_O_5_	436	0.33
30.04	Ethyl iso-allocholate	C_26_H_44_O_5_	436	0.01

The compound in the bold letter was detected at the highest percentage. Mol, molecular; (*n* = 25).

**Table 1C:** Volatile organic compounds (VOCs) obtained from the culture filtrates of *T. longibrachiatum* strain were identified by GC–MS analysis. The bold letters indicates the compound which is abundantly present in the culture filtrates of *T. longibrachiatum*.

Retention time (RT)	Compound name	Structure	MW	Abundance (%)
3.11	Tetraacetyl-d-xylonic nitrile	C_14_H_17_NO_9_	343	0.07
3.32	Tetraacetyl-d-xylonic nitrile	C_14_H_17_NO_9_	343	0.01
4.05	Tetraacetyl-d-xylonic nitrile	C_14_H_17_NO_9_	343	0.02
4.70	D-Streptamine,O-2-amino-2-deoxy-à-D-glucopyranos yl-(14)-O-[O-2,6-diamino-2,6-dideoxy -á-L-idopyranosyl-(13)-á-D-ribofurano syl-(15)]-2-deoxy-	C_23_H_45_N_5_O_14_	615	1.02
5.12	Tetraacetyl-d-xylonic nitrile	C_14_H_17_NO_9_	343	0.65
5.81	2-Myristynoyl pantetheine	C_25_H_44_N_2_O_5_S	484	3.23
6.43	Deoxyspergualin	C_17_H_37_N_7_O_3_	387	0.10
7.02	2-Myristynoyl pantetheine	C_25_H_44_N_2_O_5_S	484	0.00
7.18	Ethyl iso-allocholate	C_26_H_44_O_5_	436	0.00
7.57	Tetraacetyl-d-xylonic nitrile	C_14_H_17_NO_9_	343	0.08
8.02	2-Hexadecanol	C_16_H_34_O	242	0.51
8.21	Dodecanoic acid, 3-hydroxy-	C_12_H_24_O_3_	216	0.02
9.09	10-Octadecenal	C_18_H_34_O	266	0.01
9.76	4,5-Diamino-2-hydroxypyrimidine	C_4_H_6_N_4_O	126	3.12
10.15	Ethyl iso-allocholate	C_26_H_44_O_5_	436	0.00
10.39	Ethyl iso-allocholate	C_26_H_44_O_5_	436	0.17
10. 92	DL-Arabinose	C_5_H_10_O_5_	150	4.68
11.40	4H-Pyran-4-one, 2,3-dihydro-3,5-dihydroxy-6-methyl-	C_6_H_8_O_4_	144	2.23
12.37	Hexadecane, 1,1-bis(dodecyloxy)-	C_40_H_82_O_2_	594	1.25
**12.65**	**2-Furancarboxaldehyde,5-(hydroxymethyl)-**	**C_6_H_6_O_3_**	**126**	**68.08**
12.95	6-Acetyl-á-d-mannose	C_8_H_14_O_7_	222	1.82
13.46	à-D-Glucopyranoside, O-à-D-glucopyranosyl-(1.fwdarw.3)-á-D-fructofuranosyl	C_18_H_32_O_16_	504	1.55
14.74	Methyl 4-nitrohexanoate	C_7_H_13_NO_4_	175	3.69
15.04	Dodecanoic acid, 3-hydroxy-	C_12_H_24_O_3_	216	0.02
15.	64 Ethyl iso-allocholate	C_26_H_44_O_5_	436	0.27
16.26	à-D-Glucopyranoside,O-à-D-glucopyranosyl-(1.fwdarw.3)-á-D-fructofuranosyl	C_18_H_32_O_16_	504	5.78
17.46	Stevioside	C_38_H_60_O_18_	804	2.85
18.40	Dodecanoic acid, 3-hydroxy-	C_12_H_24_O_3_	216	0.49
20.21	3-Deoxy-d-mannoic lactone	C_6_H_10_O_5_	162	18.94
20.94	á-D-Glucopyranose, 4-O-á-D-galactopyranosyl-	C_12_H_22_O_11_	342	3.70
28.70	Ethyl iso-allocholate	C_26_H_44_O_5_	436	0.38
30.00	Ethyl iso-allocholate	C_26_H_44_O_5_	436	0.11

The compound in the bold letter was detected at the highest percentage. Mol, molecular; (*n* = 32).

### 3.3 *In vivo* planta assay

#### 3.3.1 Effect of *Trichoderma* CFs on disease severity

**Figure 3:**
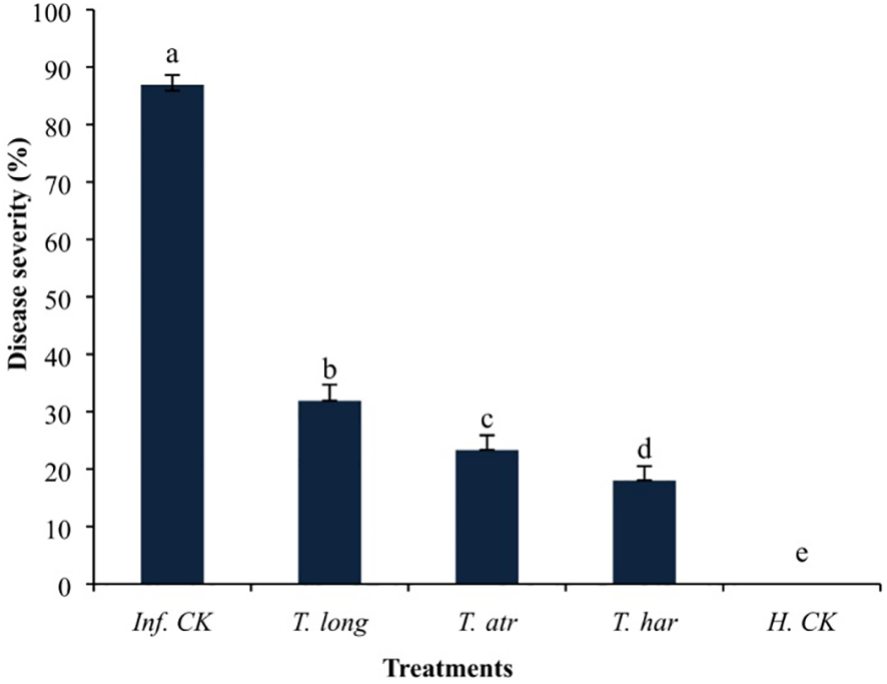
The effect of foliar application of *Trichoderma* culture filtrates (CFs) on the disease severity of *A. solani* on tomato plants in a greenhouse. Values followed by different letters indicate that means are significantly different from each other according to Fisher’s least significant difference at *p* = 0.05. *T. har*, *Trichoderma harzianum*; *T. long*, *Trichoderma longibrachiatum*; *T. atr*, *Trichoderma atroviride*; Inf. CK, infected control (pathogen inoculation); H.CK, healthy control (distilled water inoculation).

The foliar application of *Trichoderma* CFs demonstrated favorable effects on the suppression of early blight disease under greenhouse conditions. The disease severity in *T. harzianum* CF-treated plants was relatively lower (18.03%) than in *T. longibrachiatum* (31.91%) and *T. atroviride* (23.33%)-treated plants, followed by control (86.91%) (**Figure 3**). These results indicate that the foliar application of *Trichoderma* CFs drastically protected the plants from the infection of early blight pathogens under greenhouse conditions, as significant protection (79.25%) was observed in *T. harzianum* CF-treated plants, which was relatively higher than *T. longibrachiatum* (63.28%) and *T. atroviride* (73.15%) treated plants. These results illustrate that the foliar application of *T. harzianum* CFs remarkably protected the plants more than *T. longibrachiatum* and *T. atroviride* under greenhouse conditions, even after the artificial inoculation of *A. solani*. These results clearly indicate that the application of *Trichoderma* CFs has a transpicuous ability to attenuate the *A. solani* infection, and even a lower volume of CFs provided better control of the early blight pathogen.

#### 3.3.2 Effect of *Trichoderma* CFs on plant biomass

Foliar application of *Trichoderma* CFs not only alleviates the early blight infection on plants but also increases biomass, viz., the fresh and dry weight of roots and shoots. In all CF treatments, a considerable increase in the fresh and dry weight of plants was recorded. However, *T. harzianum* CFs instigated a more significant increase in fresh weight, branches, and leaves followed than other treatments (**Table 2**). In these results, *T. harzianum* CFs were found to be more responsive than *T. longibrachiatum* and *T. atroviride*. Thus, increased plant height allows more branches to emerge, which results in an increase in the weight of the plant and ultimately confers the plant growth promoter potential of these *Trichoderma* strains. Whereas no significant disparity between *T. longibrachiatum* and *T. atroviride* was recorded, although these treatments also demonstrated an increase in the growth parameters, followed by infected and healthy controls. In the present results, a significant reduction in the plant height, weight, branches, and number of leaves was recorded in the infected control (**Table 2**), which clearly demonstrates the catastrophic behavior of the early blight pathogen on plants. Results revealed that CFs of *T. harzianum* not only increase the plant height, weight, branches, and leaves of plants but also enhance the root development, which strengthens the plants to combat.

**Table 2:** Greenhouse effect of *Trichoderma* culture filtrates (CFs) on plant biomarkers post-treatment with *A. solani*. *T. atr*, *Trichoderma atroviride*; *T. har*, *Trichoderma harzianum*; *T. long*, *Trichoderma longibrachiatum*; Inf. CK, infected control (pathogen); H.CK, healthy control (water).

Treatment	No. of leaves	No. of branches	Height (cm)	Shoot weight (g)		Root weight (g)	
				Fresh	Dry	Fresh	Dry
*T. atr*	23.91 ± 1.50b	10.83 ± 1.50a	50.35 ± 2.94b	26.75 ± 1.85b	4.67 ± 0.19b	19.91 ± 0.47b	4.40 ± 0.65b
*T. har*	32.08 ± 1.10a	11.16 ± 1.10a	63.85 ± 2.20a	31.65 ± 0.47a	4.61 ± 0.22a	25.16 ± 1.10a	5.51 ± 0.37a
*T. long*	23.08 ± 1.40b	9.38 ± 1.40a	51.42 ± 2.64b	21.57 ± 0.92c	3.75 ± 0.47b	15.98 ± 2.52c	4.36 ± 0.20b
Inf. CK	15. 0 ± 0.33c	5.15 ± 0.33 b	28.42 ± 2.73d	12.82 ± 1.37e	1.68 ± 0.21d	10.98 ± 0.61d	2.31 ± 0.15d
H. CK	17.33 ± 1.23c	7.87 ± 1.23 b	34.40 ± 1.37c	16.52 ± 0.79d	2.94 ± 0.34c	12.23 ± 0.74d	3.81 ± 0.11c

### 3.4 Effect of culture filtrates on phenolic compounds

#### 3.4.1 Total phenol contents

**Figure 4:**
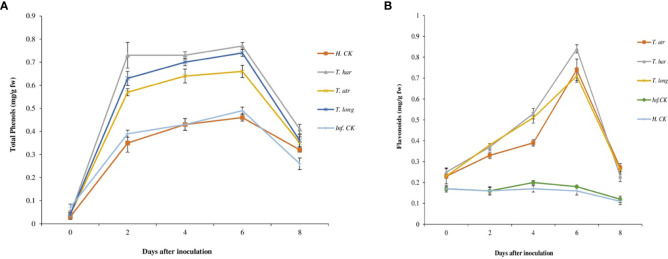
Effect of Trichoderma culture filtrates on **(A)** total phenol contents (mg gallic acid/g plant material), **(B)** flavonoids contents (mg/g plant material) in treated tomato leaves collected from greenhouse. *T. har*, *Trichoderma harzianum*; *T. atr*, *Trichoderma atroviride*; *T. long*, *Trichoderma longibrachiatum*; Inf. CK, infected control (plants treated with *A*. *solani* pathogen); H.CK, healthy control (plants treated with sterilized distilled water). Error bars on each graph represent the mean ± SE according to Fisher’s least significant difference (LSD) test at p = 0.05.

Initially, the total phenol contents (TPCs) in all treatments were constant and slightly increased after 2 days in *Trichoderma* CF-treated plants. However, this increase in TPC remained constant (0.77 mg g^−1^ plant material) until 6 days, which decreased to 0.41 mg g^−1^ plant material after 8 days of inoculation. In these results, the TPCs in *T. harzianum* CF-treated plants were significantly higher than those in *T. longibrachiatum* and *T. atroviride* (**Figure 4A**). Comparatively, the increase in TPCs in *T. harzianum* treatments (0.73–0.77 mg g^−1^ plant material) was recorded, which remained superior to other *Trichoderma* treatments. However, lower TPC in infected (0.39 mg g^−1^ plant material) and healthy control (0.35 mg g^−1^ plant material) plants were observed, which slightly increased (0.49 and 0.46 mg g^−1^ plant material, respectively) after 6 days and then declined after 8 days (0.26 mg g^−1^ plant material). These results indicate that the foliar application of *Trichoderma* CFs, particularly *T. harzianum* (in this study), escalated the phenolic compound production in tomato leaves, which perhaps activated the defense system of plants to combat *A. solani* infection.

#### 3.4.2 Flavonoids contents

Initially, the flavonoids contents were lower and constant in all treatments, including control, which showed a slight increase after 2 days (**Figure 4B**) in *Trichoderma* CF-treated plants, but no elevation in infected or healthy control plants was recorded. A continuous increase in flavonoids up to 6 days was observed, while higher flavonoids production in *T. harzianum*, *T. atroviride*, and *T. longibrachiatum*-treated plants (0.84, 0.74, and 0.71 mg g^−1^ plant material, respectively) was recorded that gradually declined after 6 days. Comparatively, *T. harzianum* demonstrated a higher and more significant increase (0.84 mg g^−1^ plant material) in flavonoids after 6 days, followed by *T. atroviride* and *T. longibrachiatum* (**Figure 4B**). Overall, *Trichoderma* treatments remained supercilious, which evidently confers the positive response of these *Trichoderma* strains on flavonoid production in tomato plants.

### 3.5 Effect of culture filtrates on antioxidant enzyme

#### 3.5.1 Phenylalanine ammonia-lyase assay

**Figure 5:**
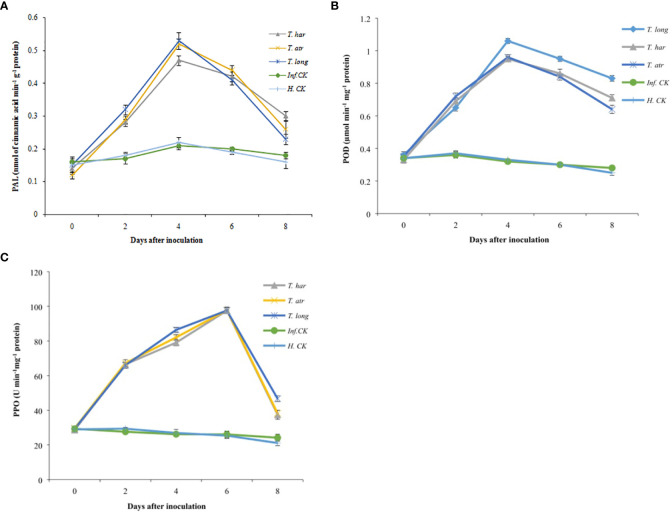
Effect of Trichoderma culture filtrates on **(A)** phenylalanine ammonia-lyase (nmol of cinnamic acid min^−1^ g^−1^ protein) activity, **(B)** peroxidase activity (µmol min^−1^ mg^−1^ protein), **(C)** polyphenol oxidase activity (U min^−1^ mg^−1^ protein) in inoculated tomato leaves collected from greenhouse. *T. har*, Trichoderma harzianum**;** *T. atr*, Trichoderma atroviride**;** *T. long*, Trichoderma longibrachiatum**;** *Inf. CK*, infected control (plants treated with A. solani pathogen); *H.CK*, healthy control (plants treated with sterilized distilled water). Error bars on each graph represent the mean ± SE according to Fisher’s least significant difference (LSD) test at p = 0.05.

Initially, in all treated plants, the phenylalanine ammonia-lyase (PAL) activity remained lower and consistent. Subsequently, a gradual increase in PAL activity was recorded after 2 days, which reached its maximum after 4 days in *T. longibrachiatum*, *T. atroviride*, and *T. harzianum* (0.53, 0.52, and 0.47 nmol of cinnamic acid min^−1^ g^−1^ protein, respectively) treated plants (**Figure 5A**). Though the PAL activity in infected and healthy control plants demonstrated a considerable increase after 4 days (0.21 and 0.22 nmol of cinnamic acid min^−1^ g^−1^ protein, respectively), this increase was not significant compared to *Trichoderma* CF-treated plants. While no significant difference between *T. longibrachiatum* and *T. atroviride*-treated plants was recorded (**Figure 5A**). On the other hand, the results herein presented concluded that the PAL activity in *T. longibrachiatum* and *T. atroviride*-treated plants was considerably higher than that in *T. harzianum*, and these *Trichoderma* strains positively affected the PAL activity in tomato plants.

#### 3.5.2 Peroxidase activity assay

Peroxidase (POD) activity in all treatments, including healthy and infected control plants, was relatively lower, and a slight increase in POD activity in *Trichoderma* CF-treated plants was observed after 2 days of inoculation (**Figure 5B**). However, the momentous increase in *Trichoderma* CF-treated plants after 4 days was recorded and remained higher in *T. longibrachiatum* (1.06 µmol min^−1^ mg^−1^ protein), followed by *T. harzianum* (0.95 µmol min^−1^mg^−1^ protein) and *T. atroviride* (0.96 µmol min^−1^mg^−1^ protein). Generally, no significant increase in POD activity in control plants was observed, though a persistent decrease was recorded. Comparatively, the POD activity in healthy and infected controls was lower than in *Trichoderma* treatments, whereas, among *Trichoderma* strains, *T. longibrachiatum* demonstrated a promising increase, which confers the positive effect of *T. longibrachiatum* on POD induction.

#### 3.5.3 Polyphenol oxidase activity

Polyphenol oxidase (PPO) activity in all treatments was lower, primarily increasing after 2 days of inoculation, and no significant difference was observed among *Trichoderma* treatments as PPO activity remained uniform (66–67.24 U min^−1^ mg^−1^ protein) but higher than healthy (29.39 U min^−1^ mg^−1^ protein) and infected control (27.62 U min^−1^ mg^−1^ protein). A significant increase in PPO activity after 4 days was recorded, with a significantly higher value after 6 days (97 ± 0.76 U min^−1^ mg^−1^ protein) and a decline after 8 days (**Figure 5C**). PPO activity in infected and healthy control plants was relatively lower than in *Trichoderma*-treated plants. In these results, all *Trichoderma* strains positively responded to significant PPO production in plants.

### 3.6 Open field trails

#### 3.6.1 Effect of culture filtrates on disease severity

**Figure 6:**
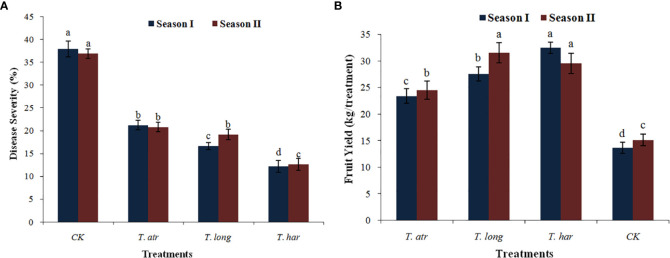
The effect of foliar application of *Trichoderma* culture filtrates (CF) on **(A)** disease severity (%) of early blight and **(B)** fruit yield in an open field under natural infection. Values followed by different letters indicate that means are significantly different from each other according to Fisher’s least significant difference at *p* = 0.05. *T. har*, *Trichoderma harzianum*; *T. long*, *Trichoderma longibrachiatum*; *T. atr*, *Trichoderma atroviride*; CK, naturally infected control (untreated plants).

The efficacy of *Trichoderma* culture filtrates (CFs) to mitigate the early blight disease under natural infection conditions was monitored, and the field application of *Trichoderma* CFs significantly reduced the early blight infection in the open field. In these results, significant reduction in disease severity (%) after the application of *T. harzianum* (SI-12.18%; SII-12.56%), followed by naturally infected control plants (SI-37.87%; SII-36.83%) in both seasons (**Figure 6A**). However, the application of *T. atroviride* and *T. longibrachiatum* also demonstrated vital control of early blight infection in both seasons (SI-21.17%; SII-20.75%) and (SI-16.62%; SII-19.12%), respectively. In the present study, the field application of *Trichoderma* CFs exhibited considerable protection to tomato plants even after natural infection with the early blight pathogen. Foliar application of *T. harzianum* CFs substantially protected the tomato plants from the natural infection of early blight disease in both seasons (SI-67.83%; SII-65.89%), while the application of *T. longibrachiatum* and *T. atroviride* CFs also provided significant protection. These results revealed that the applications of these *Trichoderma* CFs are capable of diminishing the early blight infection in open fields, and even lower volumes (50 ml/plant) can effectively control the early blight pathogen in open fields. Thus, it can be predicted that the increased volume of these *Trichoderma* CFs may provide exceptional management of this pathogen in fields. Additionally, multiple applications of CFs at different plant growth stages in a season may also help to reduce early blight infection. As in the present study, the application of these *Trichoderma* CFs in open fields provided a significant reduction in early blight.

#### 3.6.2 Effect of culture filtrates on fruit yield

The foliar application of *Trichoderma* CFs in field conditions not only curtailed the early blight disease severity but also improved the fruit yield in both seasons, as a significant increase in yield was observed. The fruit yield in *T. atroviride*, *T. longibrachiatum*, and *T. harzianum* was considerably higher than that in untreated/naturally infected control plants during season I, which increased in season II (**Figure 6B**). The *Trichoderma* treatments, viz., *T. atroviride* and *T. longibrachiatum*, exhibited lower fruit production (23.26 kg and 27.54 kg, respectively) during season I (**Figure 6B**), which increased in season II (24.48 kg and 31.54 kg, respectively). In the field application of *T. harzianum* CFs, higher (32.29 kg) fruit production was recorded in season I, which was slightly reduced in season II (29.58 kg). These results illustrate that the application of CFs of these *Trichoderma* strains in open fields not only minimized early blight infection but also improved yield, which symbolizes the positive effect of *Trichoderma* culture filtrates.

## 4 Discussion

Biological control approaches, particularly using fungal and bacterial microorganisms as biocontrol agents, are being considered as an emerging tool to combat fungicide resistance challenges. A variety of *Bacillus* and *Trichoderma* species with strong biocontrol potential, along with salt compounds and nanoparticles, have extensively been used to control plant diseases, including early blight on tomatoes (Mazrou et al., 2020; Stracquadanio et al., 2020; Castro-Restrepo et al., 2022; Imran et al., 2022b; Narware et al., 2023). Presently, the use of culture filtrates (CFs) in agriculture systems is considered an emerging method to control plant diseases and is getting a lot of attention due to its effective potential to inhibit plant pathogens. In the present study, the *in vitro* application of *Trichoderma* culture filtrates (CFs) revealed significant suppression of the mycelial growth of *A. solani*. The CFs of *T. harzianum* demonstrated strong inhibition of mycelial growth as lower mycelial growth (31.5 mm) was recorded, followed by *T. longibrachiatum* (43.06 mm) and *T. atroviride* (35.43 mm). The results herein presented illustrate the strong inhibitory potential of *Trichoderma* CFs. In a most recent study, dominant mycelial growth inhibition of *Pythium undulatum* and *Phytophthora inundata* by the CFs of *T. simmonsii* and *T. asperellum* was recorded (Ben M'henni et al., 2022). In another study, the CFs of various *Trichoderma* species, including *T. harzianum* (29.88* mm*) and *T. viride* (27.13* mm*), demonstrated remarkable suppression of *R. oryzae* (Alka and Prajapati, 2017). Additionally, the CFs of five *Trichoderma* species (*T. viride, Trichoderma* PP3, *T. harzianum*, *Trichoderma* PP2, and *T. koningii*) as biofungicides exhibited strong hindrance to *C. gloesporioides* growth (Nurbailis et al., 2019). Various studies have reported the significant mycelial growth inhibition of various fungal pathogens (Filizola et al., 2019; Mulatu et al., 2022), which sturdily confers the biocontrol potential of *Trichoderma* CFs, and these evidently support the results of the present study. *Trichoderma* species as biocontrol agents against fungal pathogens mainly involve various mechanisms, i.e., competition for food, space, and nutrients, resulting in the production of various metabolites that depreciate the mycelium of fungal pathogens (Alabouvette et al., 2006). There are ample pieces of evidence that report the abundant metabolite production by *Trichoderma* species, which showed significant inhibition of a broad spectrum of fungal pathogens from different taxonomic groups (Abbas et al., 2019; Edin et al., 2019; Mazrou et al., 2020; Mahmoud et al., 2021). In the present study, GC–MS analysis of *Trichoderma* species CFs demonstrated the abundant production of volatile organic compounds containing various classes of compounds. The chemical compounds and metabolites in the CFs of *Trichoderma* species identified from the known spectra in the National Institute of Standards and Technology (NIST) database exhibited the presence of harzianic acid (C_19_H_27_NO_6_) in *T. harzianum*, linoleic acid (C_18_H_32_O_2_) as 9,12-octadecadienoic acid (Z,Z)—in *T*. *atroviride* and hydroxymethylfurfural (C_6_H_6_O_3_) as 2-furancarboxaldehyde,5-(hydroxymethyl)—in *T. longibrachiatum* as the main components that were observed in relatively higher abundance (%). The abundance (%) and quantity of active compounds/metabolites in *Trichoderma* CFs can differ depending upon the method of compound extraction and the species of *Trichoderma*. Various researchers reported identical compounds during the GC–MS analysis of *T. harzianum*, *T. atroviride*, and *T. longibrachiatum* culture filtrates (Reithner et al., 2007; Anita et al., 2012; Stracquadanio et al., 2020), while dissimilarity in active metabolic compounds, viz., 6-pentyl-α-pyrone (Yassin et al., 2022), harzianic acid (Xie et al., 2021), acetic acid (Yassin et al., 2021), glacial acetic acid, and ethanoic acid (Siddiquee et al., 2012), was reported in the CFs of *T. harzianum* in GC–MS analysis. In the greenhouse application of CFs, *T. harzianum* demonstrated a promising effect for the reduction of disease severity that might be due to the excessive production of harzianic acid because harzianic acid is an important secondary metabolite from *T. harzianum* that has been widely reported to have strong antimicrobial and plant growth promoter potential against various phytopathogens such as *Rhizoctonia solani*, *Sclerotinia sclerotiorum*, and *Pythium irregulare* and also capable of chelating soil iron (Fe3^+^) in plants (Vinale et al., 2013; Vukelić et al., 2021; Xie et al., 2021). During GC–MS analysis, various volatile compounds, including esters, hydrocarbons, ethers, alcohols, aldehydes, ketones, and different acids, were found. Various studies reported the presence of similar compounds with considerable yield (%) differentiation in metabolites of *Trichoderma* CFs (Anita et al., 2012; Sarsaiya et al., 2020; Sornakili et al., 2020; Stracquadanio et al., 2020; Rajani et al., 2021). In greenhouses, the foliar application of *Trichoderma* CFs demonstrated a significant reduction in disease severity (%), as lower disease severity (%) was recorded in the plants inoculated with *T. harzianum* CFs (18.03%) as compared to *T. longibrachiatum* (31.91%) and *T. atroviride* (23.33%), followed by infected control (86.91%). Besides the alleviation of disease severity, the CFs of *Trichoderma* strains also exhibited a momentous increase in the biomass of plants, viz., plant leaves and branches, and the fresh and dry weight of roots and shoots, which confers the growth promoter potential of *Trichoderma* species. However, various studies reported the optimistic influence of *Trichoderma* species on plant biomass. The application of *T. harzianum* and *T. asperellum* positively increased the plant biomass of *Diplotaxis tenuifolia* (Caruso et al., 2020) and *Mentha spicata*, respectively (Castro-Restrepo et al., 2022). In another study, the combined application of *Trichoderma simmonsii* and *Aspergillus westerdijkiae* also exhibited an increase in the growth of apple trees (Ben M'henni et al., 2022). These studies sturdily confer the plant growth promoter potential of *Trichoderma* species, which strappingly supports the findings of the present study. Thus, an increase in plant biomass and reduction in disease after the application of synthetic pathogenic infection might be associated with the stimulation of antioxidants and the production of polyphenolic secondary metabolites in plants that ultimately induce resistance against pathogen infection. Secondary metabolites such as lignin and phenolic acid significantly medicate the defense activity by strengthening the rigidity of the cell wall, which eventually prevents the invasion of pathogens into plants (El-Khallal, 2007; Abo-Elyousr et al., 2008). Various pathogenesis-related (PR) proteins are coded by the host and have a significant role in the defense system of plants induced by the infection of various pathogens as well as abiotic stress (Jiang et al., 2015). In the present study, a significant increase in total phenol (TP) contents, flavonoids, phenylalanine ammonia lyase (PAL), peroxidases (POD), and polyphenol oxidase (PPO) in *Trichoderma*-treated plants was recorded that was relatively higher than untreated/control plants. However, a significant increase in TP contents was recorded after two days of inoculation, while flavonoids contents increased after 6 days of inoculation in *Trichoderma*-treated plants followed by healthy and infected controls. Flavonoids play a significant role in plants and act as detoxifying agents, phytoalexins, signal molecules, and allelochemical agents (Fawe et al., 1998; McNally et al., 2003; Peer and Murphy, 2006). The increase in TP and flavonoids in plants demonstrates that these *Trichoderma* species robustly induce systemic resistance by releasing not only proteins but also secondary metabolites. The results reported by EL-Tanany et al. (2018) and Mayo-Prieto et al. (2019) showed a considerable increase in flavonoids and total phenolic contents when the tomato plants were inoculated with *T. viride* or *T. hamatum* after the inoculation with *A. solani* and *T. velutinum* after the inoculation with *R. solani*, respectively, and these documented findings are in support of our results and strongly underpin these findings. In another study, the highest increase in enzyme activity was recorded when the tomato plants were inoculated with *T. harzianum* and *R. solani* (Youssef et al., 2016). In accordance with Mahmoud et al. (2021), tomato plants treated with *A. cerealis* and *T. harzianum* extensively increase the total phenol and flavonoid contents, and our results are in agreement with the previously reported findings and substantiate that *Trichoderma* species accumulate PR-proteins and phenolic compounds, which hinder the invasion of the *A. solani* pathogen in plants. Furthermore, tomato leaves contain solavetivone, flavonoids, lubimin, phytuberol, phytuberin, glutinosone, and rishitin, which are toxic and antimicrobial agents that act as rebellious compounds against various phytopathogens (Kim et al., 2019).

Phenylalanine ammonia-lyase (PAL), peroxidases (POD), and polyphenol oxidase (PPO) are the major enzymes for the induction of resistance in plants against biotic and abiotic stresses. Phenolic compounds are endogenous growth regulators, whereas PAL is a key enzyme for the biosynthesis of phenolic compounds from the shikimic acid pathway, which may amend the defense response by regulating the biosynthesis of phenolic compounds after different stresses (Caretto et al., 2015; Abo-Elyousr et al., 2020). The activation of PAL is mainly associated with distinct signal transduction pathways with respect to apoptotic cell death and oxidative burst (Sasabe et al., 2000), and the induction of PAL by pathogen-derived elicitors can provide significant insights into signal transduction mechanism that activates the plant response. Further, the stimulation of PAL may also enable a quantitative appraisal of both plant responses and the effectiveness of protein. Generally, tomato plants, not only fruits but roots, stems, and leaves, are also considered rich sources of antioxidants and phenolic compounds, and in this study, a significant increase in PAL contents in tomato leaves was recorded after 4 days of inoculation followed by healthy and infected control plants. A study by Kumar et al. documented that the application of *T. harzianum* and *T. viride* increased the induction of defense enzymes, including total phenol and PAL, after 48 h of inoculation against early blight infection (Kumar et al., 2022). Various studies reported a significant increase in PAL activity in tomato plants after inoculation with the suspension of *Trichoderma* species (Kashyap et al., 2020; Tripathi et al., 2021; Kumar et al., 2022), which confers the positive impact of *Trichoderma* species on plants, and these results strongly support our findings. Peroxidases (POD) widely contribute to physiological processes such as the formation of suberin and lignin phytoalexin synthesis, cross-linking of cell wall components, and/or also participate in reactive nitrogen species (RNS) and reactive oxygen species (ROS) metabolism, which activate the hypersensitive response by limiting the expansion of pathogens (Peer and Murphy, 2006). While PPO implicates the lignification of plant cells and the oxidation of polyphenols into quinines (antimicrobial compounds) during the infection of pathogens. In this study, a significant increase in POD and PPO activity after the application of *Trichoderma* CFs was recorded; thus, the increase in PPO and POD activity that restricted the pathogen growth may be due to the oxidation of phenolic compounds to quinone, which increases the antimicrobial activity and ultimately strengthens the defense system of the plant. Various researchers documented the identical findings, which showed an astronomical increase in phenolic contents in response to various treatments (Pawel et al., 2020), which affirm that the increase in phenolic compounds activates the defense system of plants against the fungal infection. It is to be believed that phytotoxic chemical compounds engender the overproduction of ROS in plants and ultimately elevate the level of phenolic compounds, which may act as sufficient antioxidants to prevent the formation of cellular deterioration due to oxidative stress. Thus, the higher activity of PPO, POD, and PAL increased the oxidation of phenolic compounds, which led to distortion of cell wall structure and restricted pathogen growth.

In the present study, the open field trials under natural infection of early blight revealed that the CFs of these *Trichoderma* strains not only mitigated the infection of the pathogen but also escalated the yield production, which epitomizes the positive effect of CFs and their capability to promote fruit production in open field conditions even during natural infection. A study by Soesanto et al. (2022) documented that the culture filtrates of *T. harzianum* not only improved the growth and yield components of tomato plants but also increased the phenolic compounds, number of fruits, and total yield under *in vivo* conditions (Soesanto et al., 2022). In another study, a significant increase in yield and growth-related parameters of *Celosia cristata* was recorded after the inoculation of *Trichoderma* strains (Wang et al., 2021). Similar findings were also reported by Alka and Prajapati (2017); Vukelić et al. (2021), and Sriwati et al. (2019) when the culture filtrates (CFs) of different *Trichoderma* strains were used against various fungal plant diseases of tomato, and these results are in agreement with the findings of the present study, which positively support these results. In this study, the beneficial effect of *Trichoderma* species was confirmed, and a safe approach using culture filtrates (CFs) to diminish the early blight disease in open fields in this region was deployed.

## 5 Conclusion

These results of the present study suggested that the foliar application of *Trichoderma* culture filtrates (CFs) considerably reduced the mycelia growth of *A. solani* under *in vitro* conditions, which confers the strong inhibitory potential of CFs against fungal pathogens. Moreover, the CFs of these *Trichoderma* strains not only decreased the early blight infection in greenhouses, but also improved plant growth-related parameters and, in field conditions, also promoted fruit production. The most important aspect of CFs is the induction of antioxidants in plants, which act as the first barrier against pathogen infection. Significantly increased antioxidant production firmly restricts the further invasion of pathogens. Therefore, an appropriate number of CFs at different growth stages of tomato plants may ameliorate plant vigor, disease severity, and fruit yield by acting as plant growth promoters. We believe that it would be of great interest to carry out identical practices in other regions to minimize the fungicide resistance risk of early blight pathogens, which are becoming more serious concerns in sustainable agricultural production.

## Data availability statement

The original contributions presented in the study are included in the article/**Supplementary Material**, further inquiries can be directed to the corresponding authors.

## Author contributions

MI: Conceptualization, experimentation, methodology, formal analysis, and writing—original draft. KA-E: Supervision and review and editing. MM: Supervision, conceptualization, formal analysis, and review and editing. MS: Supervision, revision, and formal analysis. All authors contributed to the article and approved the submitted version.
