# Supplementary figures and images for: Use of Trichoderma culture filtrates as a sustainable approach to mitigate early blight disease of tomato and their influence on plant biomarkers and antioxidants production
Source: Front Plant Sci. 2023 Jul 17;14:1192818. doi: 10.3389/fpls.2023.1192818 (PMC10388550; doi:10.3389/fpls.2023.1192818)

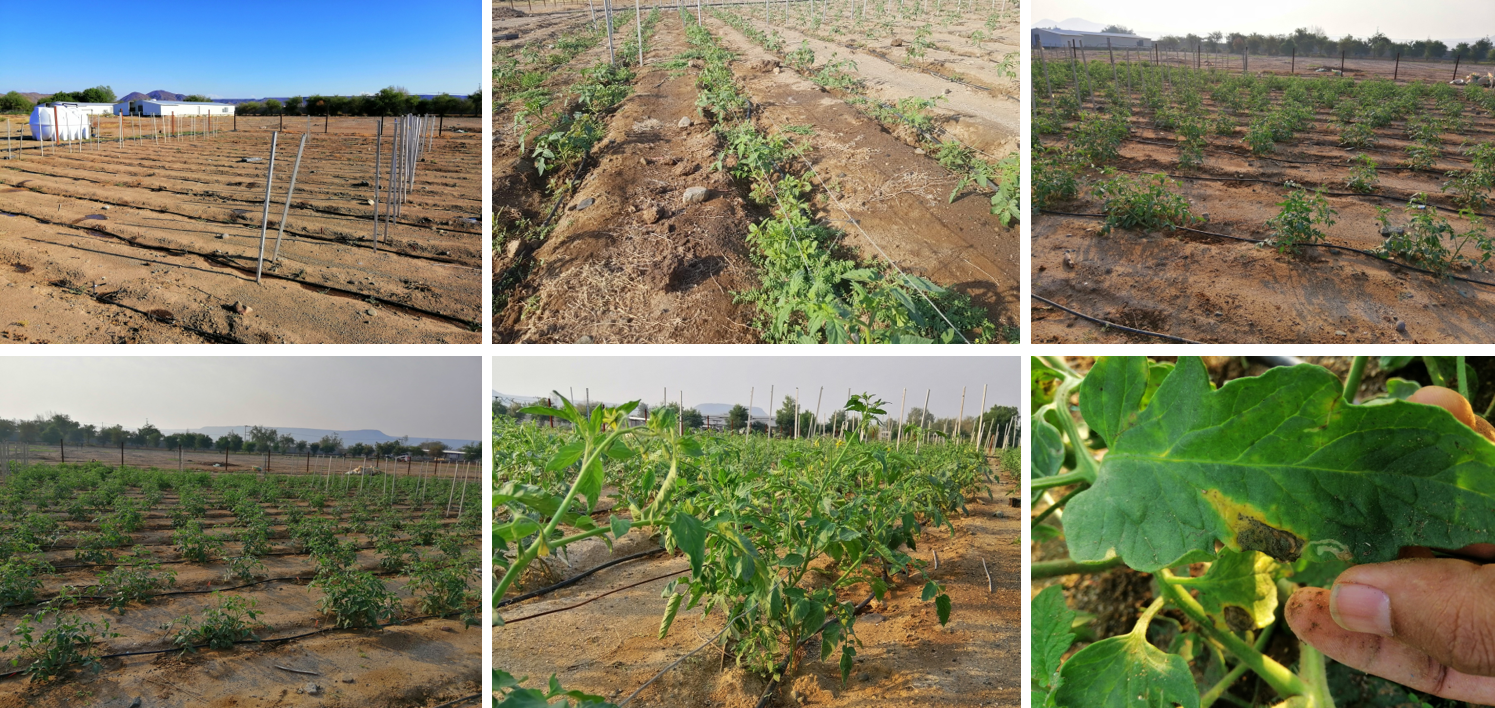

Supplement: Supplementary file 1 [file Image_1.tif]
