# Supplementary material for: Use of Trichoderma culture filtrates as a sustainable approach to mitigate early blight disease of tomato and their influence on plant biomarkers and antioxidants production
Source: Front Plant Sci. 2023 Jul 17;14:1192818. doi: 10.3389/fpls.2023.1192818 (PMC10388550; doi:10.3389/fpls.2023.1192818)
Supplement: Supplementary file 2 [file Table_1.docx]

| **Treatment** | **PAL** | | **PPO** | **POD** | **No. of leaves** | **No. of branches** | | **Height (cm)** | | | **Shoot weight (g)** | | | | | | **Root weight (g)** | | |
| --- | --- | --- | --- | --- | --- | --- | --- | --- | --- | --- | --- | --- | --- | --- | --- | --- | --- | --- | --- |
|  |  |  |  |  |  |  |  |  |  |  | **Fresh** | |  | **Dry** | | **Fresh** | | | **Dry** |
| *T. atr* | | 0.32 | 58.07 | 0.77 | 23.91 ± 1.50 b | | 10.83 ± 1.50 a | | 50.35 ± 2.94 b | 26.75 ± 1.85 b | | 4.67 ± 0.19 b | | | 19.91 ± 0.47 b | | | 4.40 ± 0.65 b | |
| *T. har* | | 0.33 | 49.82 | 0.61 | 32.08 ± 1.10 a | | 11.16 ± 1.10 a | | 63.85 ± 2.20 a | 31.65 ± 0.47 a | | 4.61 ± 0.22 a | | | 25.16 ± 1.10 a | | | 5.51 ± 0.37 a | |
| *T. long* | | 0.18 | 59.85 | 0.71 | 23.08 ± 1.40 b | | 9.38 ± 1.40 a | | 51.42 ± 2.64 b | 21.57 ± 0.92 c | | 3.75 ± 0.47 b | | | 15.98 ± 2.52 c | | | 4.36 ± 0.20 b | |
| Inf. CK | | 0.18 | 26.69 | 0.67 | 15. 0 ± 0.33 c | | 5.15 ± 0.33 b | | 28.42 ± 2.73 d | 12.82 ± 1.37 e | | 1.68 ± 0.21 d | | | 10.98 ± 0.61 d | | | 2.31 ± 0.15 d | |
| H. CK | | 0.18 | 26.36 | 0.70 | 17.33 ± 1.23 c | | 7.87 ± 1.23 b | | 34.40 ± 1.37 c | 16.52 ± 0.79 d | | 2.94 ± 0.34 c | | | 12.23 ± 0.74 d | | | 3.81 ± 0.11 c | |

Supplementary Table S1: Effect of various Trichoderma culture filtrates (CFs) on tomato Plant Biomarkers under greenhouse conditions.
